# A European Network of Email and Telephone Help Lines Providing Information and Support on Rare Diseases: Results From a 1-Month Activity Survey

**DOI:** 10.2196/ijmr.2867

**Published:** 2014-05-05

**Authors:** Francois Houyez, Rosa Sanchez de Vega, Tuy Nga Brignol, Monica Mazzucato, Agata Polizzi

**Affiliations:** ^1^European Organisation for Rare Diseases (Eurordis)ParisFrance; ^2^Federacion Espanola de Enfermedades RarasHelp Line SIOMadridSpain; ^3^Assocation Française contre les Myopathies AFM- TéléthonHelp Line MyoInfoEvryFrance; ^4^Rare Diseases Coordinating CentreRare Diseases RegistryVeneto RegionPadovaItaly; ^5^National Centre for Rare DiseasesIstituto Superiore di SanitàRomaItaly

**Keywords:** rare diseases, help line, social telephony, health services planning, health information

## Abstract

**Background:**

Information on rare diseases are often complex to understand, or difficult to access and additional support is often necessary. Rare diseases helplines work together across Europe to respond to calls and emails from the public at large, including patients, health care professionals, families, and students. Measuring the activity of helplines can help decision makers to allocate adequate funds when deciding to create or expand an equivalent service.

**Objective:**

Data presented are referred to a monthly user profile analysis, which is one of the activities that each helpline has to carry out to be part of the network. This survey aimed to explore the information requests and characteristics of users of rare diseases helplines in different European countries. Another aim was to analyze these data with respect to users’ characteristics, helpline characteristics, topics of the inquiries, and technologies used to provide information. With this survey, we measure data that are key for planning information services on rare diseases in the context of the development of national plans for rare diseases.

**Methods:**

A survey was conducted based on all calls, emails, visits, or letters received from November 1 to 30, 2012 to monitor the activity represented by 12 helplines. Data were collected by a common standardized form, using ORPHA Codes for rare diseases, when applicable. No personal data identifying the inquirer were collected. It was a descriptive approach documenting on the number and purpose of inquiries, the number of respondents, the mode of contact, the category of the inquirer in relation to the patient, the inquirer’s gender, age and region of residence, the patient’s age when applicable, the type and duration of response, and the satisfaction as scored by the respondents.

**Results:**

A total of 1676 calls, emails, or letters were received from November 1 to 30, 2012. Inquiries were mostly about specific diseases. An average of 23 minutes was spent for each inquiry. The inquirer was a patient in 571/1676 inquiries (ie, 34.07% of all cases; 95% CI 31.8-36.3). Other inquirers included relatives (520/1676, 31.03%; 95% CI 28.9-33.3), health care professionals (354/1676, 21.12%; 95% CI 19.2-23.1), and miscellaneous inquirers (230/1676, 13.72%; 95% CI 12.1-15.4). Telephone remained the main mode of contact (988/1676, 58.95%; 95% CI 56.6-61.3), followed by emails (609/1676, 36.34%; 95% CI 34.0-38.6). The three main reasons of inquiries were to acquire about information on the disease (682/2242, 30.42%; 95% CI 27.8-32.1), a specialized center/expert (404/2242, 18.02%; 95% CI 15.9-19.6), and social care (240/2242, 10.70%; 95% CI 9.1-12.0).

**Conclusions:**

The helplines service responds to the demands of the public, however more inquiry-categories could be responded to. This leaves the possibility to expand the scope of the helplines, for example by providing assistance to patients when they are reporting suspected adverse drug reactions as provided by Directive 2010/84/EU or by providing information on patients’ rights to cross-border care, as provided by Directive 2010/24/EU.

## Introduction

Rare diseases are defined as diseases affecting less than 1 in 2000 individuals in Europe or less than 200,000 people in the United States [1,2]. It is estimated that between 5000 and 8000 distinct rare diseases exist [3]. Despite their heterogeneity, rare diseases share some common features, representing a complex medical and social issue, because of their severe outcomes, considerable burden on affected individuals and their families, and impact on health services. The European Commission adopted a Communication and the Council a Recommendation on rare diseases, setting out an overall community strategy to support Member States in diagnosing, treating, and caring for citizens with rare diseases [1,4]. In both these documents, information is identified as a crucial area for action. In fact, patients with rare diseases experience an additional burden, as information on their disease can be scarce, or, when available, difficult to access or to interpret, as it is the case, for example, of information regarding genetic conditions. Health care providers as well can experience information needs, as most of them see, at most, only a few of cases in their practice.

Some studies have explored the potentialities of the use of the Internet and of the social networking to obtain information on rare diseases [5-8]. Besides the opportunities provided by these new technologies, other tools are commonly used to provide information on rare diseases, among them the telephone. Being used in the past as an efficient health communication tool, it is still widely used to provide information and support to patients affected by different conditions: cancer, HIV/AIDS, depression, etc [9-11]. Examples of helplines dealing with specific rare diseases or related problems exist, but their activities are very heterogeneous and they are not developed in the context of a harmonized framework [12-14].

The activities of 12 helplines providing information on rare diseases, mainly by telephone and email, to a broad range of users across Europe are presented. These helplines are members of the European Network of Rare Diseases Help Lines, which was created in 2006 as an outcome of the European Rapsody project [15]. To date, the helplines’ members of the network operate in 8 countries: Bulgaria, Croatia, Denmark, France, Italy, Portugal, Romania, and Spain. The network also includes helplines that are still under development in another 2 countries: Belgium and Switzerland. Data presented describe a monthly user profile analysis, which is one of the mandatory activities that each helpline has to carry out annually to be part of the network. This survey aims at exploring the information requests and the characteristics of the users of helplines set up in different European countries, active in delivering information on issues related to rare diseases.

Another aim was to analyze these data with respect to users’ characteristics, helplines’ characteristics, topics of the inquiries, and technologies used to provide information. Measuring the activity of existing helplines can help decision makers to allocate adequate funds when deciding to create or expand an equivalent service. Helplines are compared according to their nature (type of organization, eg, patients’ organization or governmental service), their scope (all rare diseases, or a specific subgroup), their composition (run by volunteers and/or paid staff), and their mode of operations (via telephone and/or emails).

With this survey, we measure the actual activity of 12 helplines, and these data are key for planning information services on rare diseases in the context of the development of national plans for rare diseases before the end of 2013, as recommended by the Council of the European Union [4] and a Commission Communication on Rare Diseases [1].

## Methods

### The Survey

The survey characterizes who the inquirers were, why they were contacting the helplines, about which diseases, and which responses they received; a total of 13 data were collected for each call, email, or other. Interoperability was ensured by using the ORPHA codes to share information on the diseases for which inquirers contact the helplines [16]. ORPHA codes refer to the Orphanet classification of diseases.

For the survey, all helplines were supplied with the same standardized form to fill in with their data. All fields had been agreed and tested. All helplines filled in the survey based on the totality of inquiries received in November 2012. Data from the whole survey are presented here. Details on the types of data collected ar found in Multimedia Appendix 1.

### Ethical Approval: Compliance With Data Protection

Demonstration of compliance with national legislation on data privacy protection was mandatory to become a full network member. Helplines applying to the network documented their registration to the national regulatory authority in writing.

As this survey was a descriptive and anonymized analysis of the inquiries received, it was not necessary to seek prior authorization from an ethics committee. Data identifying the participating individuals were not shared. Demographic data used in this analysis only included age range (and not the exact date of birth), gender, category, and region of residence. None of the demographic data collected could lead to an inquirer’s identification.

### Description of Participating Helplines

Each helpline was responsible for its own funding; some benefited from public grants or donations but their funding was often fragile and their sustainability was challenged.

Various operating modes could be observed among these helplines according to their specific characteristics (information for the Danish helpline VISO [National Organization for Knowledge and Specialist Consultancy] was excluded as its administrative status was changing): nature, composition, mode of operation, cost structure, and scope.

Nature was defined as being governed by a patient-driven organization (seven helplines) or by health care professionals/government organization. Helplines governed by health care professionals or by the government were grouped together as they represented four helplines (governed means the administration of the service from the legal point of view; not known for the Danish helpline). Composition referred to the type of respondents who could be either paid staff only (7/12, 58%), by volunteers only, or by a mix of volunteers and paid staff (4/12, 33%; not known for the Danish helpline). Regarding the mode of operation, there was no mutually exclusive mode of operation, as all helplines except one received inquiries both by telephone and by emails. However, some were contacted by phone for one-half or more of their inquiries (6/12, 50%), others were more often contacted by email (6/12, 50%), depending on how helplines advertise their telephone number or email address, and on the inquirers’ choice. The cost structure showed that nine helplines charged a local call or full call to phone inquirers, two were offering free of charge call service (not shown; not known for the Danish helpline). The scope pointed out that nine helplines were providing information on all rare diseases, three focused on one rare disease, or a group of rare diseases (congenital anemia, neuromuscular disorders, and myasthenia gravis). Other characteristics were not considered for this analysis (hours of operation, resources for service awareness campaigns, date of creation, etc).

### Variables in the Standardized Form

Possible responses were agreed upon by helplines prior to the survey. These responses are outlined in Textbox 1.

**Possible responses by helplines.**

- Category of the inquirer, his/her gender, and age: a patient, a relative, a friend, a partner, a health care professional, a media (information professionals) , a student, a member of a patients’ organization, or not specified.
- Inquirer’s region of residence: this data has been recorded but is not presented in this article.
- Duration of the inquiry: for calls, respondents were requested to estimate the duration of the calls and for emails, respondents were requested to estimate the duration of the time needed to read the inquiry, to draft and validate the response.
- Purpose of the contact: information on disease, information on a specialist or center, contact with other patient, support, information on social care, obtaining exemption for full reimbursement, information on a patients’ organization, follow-up, sign posting, other or not specified, information on events.
- Disease: helpline respondents were asked to use the Orpha codes when the diagnosis was known to the inquirer, else an organ class could be documented or case classified as “undiagnosed.”
- How the inquirer heard about the helpline.
- Response: it relates directly to the purpose of the inquiry. However, the helpline respondent may provide additional information based on his/her own evaluation of what the inquirer may need to know, even if the inquirer did not spontaneously asked for this information. Several responses could be given.
- Satisfaction: the satisfaction was scored by each respondent on a subjective satisfaction scale from 0 to 10, 10 corresponding to the highest satisfaction for the handling of the inquiry.

## Results

### Overview

A total of 1676 inquiries were received during November 2012, ranging from 3 to 389 per helpline (average 139.7; 95% CI 66.0-213.3). During this period, 51 respondents (paid staff or volunteers) answered the inquiries, for an average of 33.9 inquiries per respondent, ranging from 1.5 to 97.3 (95% CI 13.5-52.2). This represented a large diversity between helplines and can be explained partially by the age of the helpline, by their respective advertisement resources to make the service known to their respective publics. Information on the existence of the helpline was found on the Internet (317/1169, 27.12%; 95% CI 24.6-29.7), through health care professionals (284/1169, 24.29%; 95% CI 21.8-26.8), media (182/1169, 15.57%; 95% CI 13.5-17.7), or other means (including patients’ organizations 127/1169, 10.86%; 95% CI 9.1-12.7; Table 1). Telephone and emails represented 95.29% (1597/1676; 95% CI 94.3-96.3) of methods used to contact a helpline, and the telephone was the most frequent (988/1676, 58.95%, 95% CI 56.6-61.3; Table 2).

**Table 1:** Distribution of diseases.

Type of diseases	All helplines		Specialized helplines excepted	
	n	%	n	%
Malignancies	42	3.0	42	3.0
Cognitive/neurological disorders	535	38.7	319	22.9
Sexual abnormalities	24	1.7	24	1.7
Skin, tooth diseases	70	5.1	70	5.0
Musculoskeletal	148	10.7	113	8.1
Hematology	89	6.4	87	6.3
GI track, liver, kidney	63	4.6	63	4.5
Inborn errors of metabolism/endocrine disorders	116	8.4	107	7.7
Cardiovascular, respiratory	84	6.1	81	5.8
Eye/vision	63	4.6	61	4.4
Others	192	13.9	188	13.5
Total	1426	100	1155	100

**Table 2:** Number of inquiries per helpline by phone, through a helpline from a health care professional, or through a patients’ organization.

Name of helpline	Total number of inquiries, n	Number of respondents, n	Phone, n (%)	Helpline, n (%)	Patients’ organization (%)
AFM Téléthon^a^	254	16	120 (47.2)	39 (15.4)	79 (31.1)
CVRR^b^	294	6	227 (77.2)	222 (75.5)	19 (6.5)
Croatian Help Line	15	3	9 (60.0)	0 (0.0)	3 (20.0)
ENERCA^c^	3	2	0 (0.0)	0 (0.0)	1 (33.3)
ICRDOD^d^	17	2	11 (64.7)	2 (11.8)	4 (23.5)
Linha Rara	196	3	62 (31.6)	6 (5.6)	8 (7.4)
MRIS^e^	389	4	262 (67.4)	8 (2.1)	15 (3.9)
Myasthenia Gravis MGR	28	2	10 (35.7)	3 (10.7)	15 (53.6)
NORO^f^Help Line	90	1	30 (33.3)	1 (1.1)	21 (23.3)
SIO-FEDER^g^	203	6	74 (36.5)	0 (0.0)	0 (0.0)
TVMR^h^	170	3	169 (99.4)	2 (1.2)	0 (0.0)
VISO^i^	17	3	14 (82.4)	1 (5.9)	7 (41.2)
Total	1676	51	988 (58.9)	284 (24.3)	172 (14.7)

^a^Association Française contre les Myopathies-Téléthon

^b^Coordinating Centre Veneto Region

^c^European Network for Rare Congenital Anaemia

^d^Information Centre for Rare Diseases and Orphan Drugs

^e^Maladies Rares Info Services

^f^Norwegian-Romanian Information Centre

^g^Information and Orientation Service of the Spanish Federation of Rare Diseases

^h^Telefono Verde Malattie Rare

^i^National Organization for Knowledge and Specialist Consultancy, Denmark

There were a total of 1426 inquiries on the various diseases from all helplines. From these inquiries, 37.66% (537/1426) discussed distinct diseases or groups of diseases. When the disease was identified and coded, the largest class of rare diseases inquired for was cognitive/neurological disorders (535/1426, 37.52%), followed by musculoskeletal disorders (148/1426, 10.39%), reflecting the presence of two helplines specialized in neuromuscular diseases (Association Française contre les Myopathies-Téléthon [AFM-Telethon] and Myasthenia Gravis Romania), as shown in Table 1.

Among these 537 diseases, 95 (17.7%) were very rare diseases with less than 4500 patients in the European Union and the prevalence was unknown for 37.1% (199/537) of them, indicating very little information available. The threshold of 4500 patients corresponds to a disease prevalence of 1 in 100,000 inhabitants in the European Union. A significant number of diseases (49/537, 9.1%) were not rare diseases (prevalence >5/10,000 inhabitants in the European Union or >250,000 cases in the European Union; Table 3). In such a case, most of the helplines could still respond to the inquirer.

**Table 3:** Number of diseases by prevalence.

Prevalence range		Number of diseases	%
<1/1000000	>500 patients	58	10.8
1-9/1000000	500-4500	37	6.9
1-9/100000	5000-45,000	102	19.0
1-5/10000	50,000-250,000	59	11.0
6-9/10000	300,000-450,000	8	1.5
>1/1000	<500,000	41	7.6
Unknown prevalence		199	37.1
Group of diseases		32	6.0
Total		536	100.0

Globally, the number of enquiries by respondent was manageable (average 32.9, ranging from 1.5 to 97.3; 95% CI 13.5-52.2). No helpline was saturated (however, not all respondents were working full time to respond to inquiries). The duration of inquiries was on average 23 minutes (median 15; 95% CI 9.3-36.4) and the distribution is shown in Table 4. Inquiries needing more than 20 minutes represented 41.54% (604/1454) of all inquiries. There were 537 different rare diseases discussed, a majority of which are very rare and with very little information available.

**Table 4:** Distribution of the duration of inquiries.

Duration range (minutes)	Number of inquiries
1-4	41
5-7	160
8-9	111
10-14	289
15-19	253
20-24	217
25-34	153
35-59	108
60-89	69
90-119	27
≥120	30
Total	1458

### Analysis of Inquiries According to Helplines’ Characteristics

#### Nature of the Helpline

Health care professionals tended to contact helplines that were more often driven by other health care professionals than helplines driven by patients. Of the 484 inquiries to helplines driven by health care professionals/governmental authority, 42.4% (205/484; 95% CI 35.6-49.1) were from professionals, versus 12.27% (144/1174; 95% CI 6.9-17.6) for inquiries to helplines driven by patients. Of the 349 professionals who contacted a helpline during the period, 58.7% (205/349; 95% CI 53.6-63.9) contacted a helpline driven by their colleagues or governmental authorities, and 41.3% (144/349; 95% CI 36.1-46.4) contacted a helpline driven by patients (Table 5).

**Table 5:** Inquirer category according to the helplines’ characteristics (VISO, Denmark excluded).

Variables	Nature						Scope						Composition					
	Patient-driven (SIO-Feder^a^, AFM Telethon^b^, Linha Rara, NORO^c^, MGR^d^, MRIS^e^, Croatian HL)			Health care professional/ governmental (ENERCA^f^, ICRDOD,^g^CCVR^h^, TVMR^i^)			General (TVMR, CCVR, MRIS, SIO-FEDER, Croatian HL, NORO, Linha Rara, VISO, ICRDOD)			Specific (AFM Téléthon, MGR, ENERCA)			General (TVMR, CCVR, MRIS, SIO-FEDER, Croatian HL, NORO, Linha Rara, VISO, ICRDOD )			Specific (AFM Téléthon, MGR, ENERCA)		
Inquirer’s category	n	%	95% CI	n	%	95% CI	n	%	95% CI	n	%	95% CI	n	%	95% CI	n	%	95% CI
Patient	437	37.2	32.7-41.8	126	26.0	18.4-33.7	467	33.6	29.3 -37.9	104	36.5	27.2-45.7	426	33.5	29.1-38.0	137	35.2	27.2-43.2
Relative, parent	396	33.7	29.1-38.4	121	25.0	17.3 - 32.7	457	32.9	28.5 -37.2	64	22.5	12.2 - 32.7	393	30.9	26.4-35.5	124	31.9	23.7-40.1
Health care professional	144	12.3	6.9-17.6	205	42.4	35.6 - 49.1	289	20.8	16.1 -25.5	65	22.8	12.6 -33.0	281	22.1	17.3-27.0	68	17.5	8.5-26.5
Student	18	1.5		2	0.4		16	1.2		4	1.4		15	1.2		5	1.3	
Friend, partner	49	4.2		10	2.1		54	3.9		5	1.8		53	4.2		6	1.5	
Patient organization	37	3.2		17	3.5		25	1.8		29	10.2		21	1.7		33	8.5	
Media	1	0.1		0	0.0		1	0.1		0	0.0		1	0.1		0	0.0	
Not specified/Unknown	92	7.8		3	0.6		83	6.0		14	4.9		80	6.3		16	4.1	
Total	1174			484			1391			285			1270			389		

^a^Information and Orientation Service of the Spanish Federation of Rare Diseases

^b^Association Française contre les Myopathies-Téléthon

^c^Norwegian-Romanian Information Centre

^d^Myasthenia Gravis Romania

^e^Maladies Rares Info Services

^f^European Network for Rare Congenital Anaemia

^g^Information Centre for Rare Diseases and Orphan Drugs Bulgaria

^h^Coordinating Centre Veneto Region Italy

^I^Telefono Verde Malattie Rare Italy

Inquiries lasted longer for helplines driven by patients (23.7 minutes; 95% CI 22.2-25.3) versus helplines driven by health care professionals/governmental authority (19.7 minutes; 95% CI 17.8-21.6), median of 15 minutes for both (Table 6). The satisfaction as scored by respondents themselves was also different depending on the nature of the helpline. They were more satisfied in helplines driven by patients but the difference was small (9.07/10 [95% CI 8.98-9.16] vs 8.78/10 [95% CI 8.65-8.9]; Table 6).

Regarding the purpose of the inquiry, the only difference was for inquiries to obtain “exemption,” for instance when the helpline was driven by health care professionals/governmental authorities, the inquiries were more likely to ask questions about exemption in the form of reimbursement of care (158/745, 21.2% [95% CI 14.8-27.6] vs 14/1515, 0.92% [95% CI 0.0-5.9]; Table 7).

Responses given differed by nature of helplines. Patient-driven helplines tended to be more likely to provide psychological support (100/1900, 5.26% [95% CI 0.9-9.6] vs 6/930, 0.6% [95% CI 0.0-7.1]) but the difference is not statistically significant, and helplines run by health care professionals/governmental authority were more likely to provide information on access to treatment (215/930, 23.1% [95% CI 17.5-28.8] vs 27/1900, 1.42% [95% CI 0.0-5.9]), or to orientate to an expert (178/930, 19.1% [95% CI 13.4-24.9] vs 169/1900, 8.89% [95% CI 4.6-13.2]; Table 8).

**Table 6:** Duration of inquiries and satisfaction according to the helplines’ characteristics.

		Nature (VISO^a^in Denmark excluded)			Scope				Composition (VISO in Denmark excluded)		
		Patient-driven	Health care professionals / governmental	Total	All RD^b^	Specific diseases	Total		Paid staff only	Others	Total
**Durations (minutes)**											
	Average (95% CI)	23.7 (22.2-25.3)	19.7 (17.8-21.6)	22.4 (21.2-23.7)	24 (22.6-25.4)	17.9 (15.5-20.3)	22.8 (21.6-24.0)		24.7 (23.1-26.2)	16.3 (14.9-17.7)	22.4 (21.2-23.7)
	Median								15	14.5	15.0
	n	969	469	1438	1172	283	1455		1052	386	1438
**Satisfaction**											
	Average (95% CI)	9.07 (8.98- 9.16)	8.78 (8.65-8.9)	8.97 (8.9-9.04)	9.03 (8.9-9.1)	8.85 (8.7-9.0)	8.97 (8.9-9.0)		8.93 (8.8-9.0)	9.02 (8.9-9.1)	8.97 (8.9-9.0)
	Median	9	9		9	9			9	9	
	n	580	302	882	615	284	899		496	386	882

^a^National Organization for Knowledge and Specialist Consultancy

^b^RD: rare diseases

**Table 7:** Purpose of the inquiry, the responses given, and the nature of the helplines.

Variable		Patient-driven			Health care professionals or governmental		
		n	%	95% CI	n	%	95% CI
**Purpose**						
	Information on disease	495	32.67	28.5-36.8	184	25.70	18.5-30.9
	Specialist/center	258	17.03	12.4-21.6	142	19.06	12.6-25.5
	Contact with other patient	68	4.49		8	1.07	
	Support	99	6.53	1.7-11.4	30	4.03	
	Social care	177	11.68	7.0-16.4	58	7.79	0.9-14.7
	Exemption	14	0.92	0.0-5.9	158	22.21	14.8-27.6
	Patients’ organization	105	6.93	2.1-11.8	9	1.21	
	Follow-up	75	4.95	0.0-9.9	21	2.82	
	Sign-posting	25	1.65		11	1.48	
	Events	28	1.85		9	1.21	
	Other	171	11.29	6.5-16.0	84	11.28	8.7-2.19
	Blank	0	0.00		1	0.13	
	Total	1515			745		
**Response given**						
	Provide contact with relevant organization	347	18.26	14.2-22.3	141	15.16	9.2-21.1
	Provide information on how to create an organization	2	0.11		2	0.22	
	Provide info on disease and care	434	22.84	18.9-26.8	161	17.31	11.5-23.2
	Provide information on scientific literature and research	60	3.16		18	1.94	
	Legal advice	18	0.95		7	0.75	
	Orientation to expert	169	8.89	4.6-13.2	178	19.14	13.4-24.9
	Provide information on access to treatment and regulatory affairs	27	1.42	0-5.9	215	23.12	17.5-28.8
	Provide information on cross border care	6	0.32		4	0.43	
	Psychological support	100	5.26	0.9-9.6	6	0.65	0.0-7.1
	Provide info on disability/social rights	120	6.32	2.0-10.7	139	14.95	9.0-20.9
	Contact with other patient	57	3.00		0	0.00	
	Provide information on clinical trials and registries	9	0.47		13	1.40	
	Provide information on respite care	10	0.53		1	0.11	
	Provide info on events	21	1.11		6	0.65	
	Follow-up	82	4.32		4	0.43	
	Link to Orphanet or other sites	193	10.16	5.9-14.4	6	0.65	0.0-7.1
	Blank or other actions	245	12.89	8.7-17.1	29	3.12	0.0-9.4
	Total	1900			930		

**Table 8:** Purpose of the inquiries, responses given, and the scope of the helpline.

		Scope					
		All rare diseases			Specific ones		
		n	%	95% CI	n	%	95% CI
**Purpose**							
	Information on disease	542	29.01	25.2-32.8	140	34.15	26.3-42.0
	Specialist/center	364	19.48	15.4-23.6	40	9.75	0.6-19.0
	Contact with other patient	59	3.16		18	4.39	
	Support	88	4.71	0.3-9.1	41	10.00	0.8-19.2
	Social care	213	11.40	7.1-15.7	27	6.58	0.0-15.9
	Exemption	172	9.21	4.9-13.5	0	0.00	
	Patients’ organization	104	5.57		10	2.44	
	Follow-up	50	2.68	0.0-7.2	46	11.22	2.1-20.3
	Sign posting	21	1.12		19	4.63	
	Events	12	0.64		25	6.10	
	Other	242	12.96	8.7-17.2	44	10.73	1.6-19.9
	Blank	1	0.05		0	0.00	
	Total	1868			410		
**Response given**							
	Provide contact with relevant organization	403	16.36	12.7-20.0	88	22.98	14.2-31.8
	How to create an organization	3	0.12		1	0.26	
	Provide info on disease and care	510	20.70	17.2-24.2	86	22.45	13.6-31.3
	Provide information on scientific literature and research	35	1.42		44	11.49	
	Legal advice	26	1.06		4	1.04	
	Orientation to expert	296	12.01	8.3-15.7	53	13.84	4.5-23.1
	Access to treatment and regulatory affairs	241	9.78	6.0-13.5	2	0.52	0.0-10.5
	Provide information on cross border care	6	0.24	4	4	1.04	
	Psychological support	78	3.17	29	29	7.57	
	Provide info on disability/social rights	254	10.31	6.6-14.0	7	1.83	0.0-11.8
	Contact with other patient	53	2.15		4	1.04	
	Provide information on clinical trials and registries	18	0.73		4	1.04	
	Provide information on respite care	10	0.41		1	0.26	
	Provide info on events	19	0.77		8	2.09	
	Follow-up	76	3.08		10	2.61	
	Link to Orphanet or other sites	194	7.87	4.1-11.7	6	1.57	0.0-11.5
	Blank or other actions	242	9.82		32	8.36	
	Total	2464			383		

#### Composition of the Service

Patient-driven helplines could be employed by paid staff only (Information and Orientation Service of the Spanish Federation of Rare Diseases [SIO-Feder], Croatian helpline), volunteers only, or a mix of paid staff and volunteers (Linha Rara, Maladies Rares Info Services [MRIS] , AFM-Téléthon, Myasthenia Gravis Romania, Norwegian-Romanian Information Centre [NORO] helpline), whereas most helplines run by health care professionals or governmental authorities employed paid staff only (Coordinating Centre Veneto Region, European Network for Rare Congenital Anaemia [ENERCA], Telefono Verde Malattie Rare), and only one operated with a mix (Information Centre for Rare Diseases and Orphan Drugs [ICRDOD]).

Helplines operated by paid staff only had the largest proportion of inquirers who were health care professionals (281/1270, 22.13% [95% CI 17.3-27.0] vs 68/389, 17.5% [95% CI 8.5-26.5] for other helplines), but this was not statistically significant (Table 5). They also had the longest duration of the inquiries at 24.7 minutes [95% CI 23.1-26.2] versus 16.3 minutes [95% CI 14.9-17.7] for other helplines (Table 6). They had the same level of satisfaction for the response given/handling of the inquiry compared with other helplines (8.93 [95% CI 8.8-9.1] vs 9.02 [95% CI 8.9-9.1]; Table 6).

#### Scope

There were important differences in the activity of helplines according to their scope (Table 6). The duration of the inquiry was longer for general diseases helplines (24 minutes, 95% CI 22.6-25.4 vs 17.9 minute, 95% CI 15.5-20.3; Table 6).

Main differences occurred for the purpose of the inquiry. For general helplines, to identify a specialist, inquiries were more frequent but not statistically significant compared with disease-specific helplines (364/1868, 19.49% [95% CI 15.4-23.6] vs 40/410, 9.8% [95% CI 0.6-19.0]), which was similar to inquiries about social care (213/1868, 11.40% [95% CI 7.1-15.7] vs 27/410, 6.6% [95% CI 0.0-15.9]). The difference was clear for exemption, or reimbursement of care (172/1868, 9.21% [95% CI 4.9-13.5] vs 0/410). Conversely, inquiries to obtain support were more frequent to disease-specific helplines (41/410, 10.0% [95% CI 0.8-19.2] vs 88/1868, 4.71% [95% CI 0.3-9.1]), which was similar to follow-up inquiries (46/410, 11.2% [95% CI 2.1-20.3] vs 50/1868, 2.68% [95% CI 0.0-7.2]) but was not statistically significant (Table 8). Regarding the responses given, there was no difference by scope of the helplines (Table 8).

### Results According to the Mode of Contact, Telephone Versus Emails

Table 9 shows that the number of male inquirers who used telephone were slightly higher than those who used email, although this difference was not statistically significant. There were 28.3% (280/988, [95% CI 23.1-33.6]) males who preferred the telephone compared with 25.2% (153/607, [95% CI 18.3-32.1]) who would have rather used email.

**Table 9:** Differences in inquiries based on how the inquirer contacted the helpline (telephone or email).

Variables		Contact mode					
		Phone			Emails		
		n	%	95% CI	n	%	95% CI
**Inquirer's gender**							
	Male	280	28.34	23.1-33.6	153	25.21	18.3-32.1
	Female	702	71.05	67.7-74.4	439	72.32	68.1-76.5
	Unknown	6	0.61		15	2.47	
	Total	988			607		
**Satisfaction**							
	Average	8.72		8.6-8.8	8.90		8.7-9.1
	SD	1.25			1.95		
	N	482			364		
**Purpose**							
	Information on disease	388	27.02	22.6-31.4	264	32.80	27.1-38.5
	Specialist/center	241	16.78	12.1-21.5	126	15.65	9.3-22.0
	Contact with other patient	22	1.53		48	5.96	
	Support	82	5.71	0.7-10.7	32	3.98	
	Social care	134	9.33	4.4-14.3	64	7.96	1.3-14.6
	Exemption	148	10.31	5.4-15.2	24	2.98	
	Patients’ organization	70	4.87		36	4.47	
	Follow-up	63	4.39		43	5.34	
	Sign-posting	29	2.02		25	3.11	
	Events	41	2.86		17	2.11	
	Other	218	15.18	10.4-19.9	126	15.65	9.3-22.0
	Total	1436			805		

### Relevance of Responses Provided by Helplines

For inquiries about information on a disease, specialists or experts, contact with other patients, and for social care, we analyzed the exact match between the request and the response given calculated as the proportion between the purpose of the inquiry and the relevant response given. For 1574 inquiries about information, specialists, contact with other patients, social care, or exemption, a response could be given to 1173 for a satisfaction rate of 74.52% (1173/1574; Table 10).

When the purpose of the inquiry was to obtain information on the disease, 68.1% (464/681) of responses contained information on the disease. For the remaining requests for information, 28.3% (193/681) the respondents could redirect the inquirer to a more specific information source (Table 10).

When the purpose of the inquiry was to identify a specialist/expert, 62.1% (251/404) of inquiries were satisfied, and when the purpose was to establish contact with another patient, 44% (34/77) were satisfied. When the purpose was to obtain information on social care, 45.4% (109/240) of the inquiries were satisfied. Lastly, when the purpose was to obtain information on exemption for full coverage of care expenses, 55.2% (95/172) of inquiries were satisfied (Table 10). Of note, when the response given did not exactly match the question, helplines could redirect the inquirer to another service or source of information in most cases.

**Table 10:** Correlation between purpose of inquiry and responses given.

Purpose of enquiry	Asked for, n	Responded, n	% match between “asked” and “responded to”
Information on disease	681	657	96.5
Specialist/expert	404	251	62.1
Contact with other patient	77	61	79.2
Social care	240	109	45.4
Exemption^a^	172	95	55.2

^a^To obtain full reimbursement of care

## Discussion

### Significance of the Study

The issue of information is crucial, especially when dealing with rare diseases. In this field, the need for reliable and validated information is equally strongly perceived by patients, their relatives, and health professionals. To the best of our knowledge, this is the first study focusing on the activity measurement of a network of helplines active in rare diseases. Even if individual helplines conduct their own activity and satisfaction surveys, these surveys are rarely published.

The 12 participating helplines diverge in their nature, composition, operation mode, and scope. Some are managed by patients’ organizations, others by health care professionals or governmental organizations; some employ paid staff only, others volunteers only, and others a mix of paid staff and volunteers; some operate mostly by telephone, others mostly by email; some are addressing all rare diseases, and others a single or a group of rare diseases. One question was to explore whether this was reflected in the category of inquirers, in the type of questions helplines receive, in the type of answers they provide, in the duration of calls/emails, or in the diseases inquired. In other words, whether the service differed given the type of helpline. Overall, despite some differences, these factors do not influence significantly the service provided by the helplines. They may differ greatly in terms of structure, governance, composition, or specificity, but the service provided to the inquirers is of same quality.

By providing quantitative information across a range of important variables, our survey showed that these helplines, although different in language and location, can work together and collaborate. They can exchange data that document on their overall activity, and focus where the needs are. A priority is to provide information on very rare diseases, to help patients identify a specialist or a specialized center, or to address social issues. These findings are consistent with the results of the EurordisCare3 survey conducted in 16 European countries, which documented difficulties in accessing specialized centers for rare diseases, and the need for more information on social services [17].

For the collection of information on the diseases inquired, it was important to implement the use of Orpha Codes by all helplines. An Orpha code is unique identifying number assigned by Orphanet to a given disease or a group of diseases. Orphanet is the reference portal for information on rare diseases and orphan drugs, for all audiences [16]. Compared with the Call Profile Analysis conducted in 2009 and 2011, 10 of 12 helplines used the Orpha codes in 2012, compared with 7 of 11 helplines in 2011 and to 3 of 8 in 2009, ensuring more complete information on the diseases. A large part of the inquiries related to rare neurological/cognitive disorders (536/1445, 37.09%). The need for information on these disorders has been reported [18] and reflects their considerable burden on patients and families.

The telephone was the most frequent method used to contact a helpline (988/1676, 58.95%). This was also the case in previous Caller Profile Analysis performed in 2011 and 2009 (not shown), and this figure is stable. We showed the comparison between telephone and emails, and both methods will continue to co-exist; despite the increasing use of the new technologies as sources of health-related information [19,20], people still value and consult more traditional information sources [21,22]. This seems to be the case also for rare diseases. The method for contacting the helpline service (telephone vs email) did not differ by inquirers’ category, except for patients who tended to use the telephone more and for students who tended to use the email more. A confounding factor could be the age, with a trend for the youngest inquirers to use email more often, but there were too few inquirers of 19 years of age or below to do this analysis.

As no major difference exists among helplines according to their nature, scope, or composition, we cannot recommend one type of helpline compared with another. The respective roles of helplines run by patients or by health care professionals appear complementary, for example, the former providing more often psychological support or contact with another patient or an association, and the latter providing more often information to obtain full coverage of care by health insurance or information on treatments and regulatory affairs.

Recommendations for funding is based on the average duration of inquiries, on the complexity of finding accurate medical information, and range of possible purposes. Also, it seems a 1.5 full-time staff is needed to start operating the service, for an annual budget of €150,000 to €300,000, according to average European salaries, including training costs both for staff and volunteers and service quality assurance.

### General Limitations and Assumptions

The helplines that are member of the European Network of Rare Diseases Helplines are very heterogeneous. In particular, their monthly activity varies greatly (in our survey conducted in November 2012 it ranged from 3 inquiries to 389). Helplines with highest numbers of respondents were organized in a national or regional way: national ones are Maladies Rares Info Services in France and the Coordinating Centre for Rare Diseases of the Veneto Region, and regional ones with AFM-Téléthon with respondents at the headquarters and in each of their 25 regions services, and with SIO-Feder with 6 respondents in 5 regions.

Although an 11-month survey is questionable in terms of duration and outcome measures, November might be considered as a representative month. There was no special rare disease-related event in any of the participating countries that could affect the number of inquiries. For example, the annual fund raising event “Téléthon” in France and Italy takes place at the beginning of December, and for 36 hours the public number of Maladies Rares Info Services is displayed on several television channels and broadcasted on radios. Other national events take place in other periods, except in November.

Nevertheless, in this attempt to compare the activity and the service provided by helplines that differ greatly in their nature, composition, scope, and cost structure, a main limitation was the absence of real choice for the inquirers (eg, in no country was there the choice between a patients-driven or a health care professionals/governmental authority-driven helpline). Even if the inquirer could always contact the helpline by telephone or by email, this was in fact determined by the respective publicity of the telephone number or email address.

One outcome measure would have consisted in analyzing the inquirers’ satisfaction. For the time being, this information is not collected by the helplines, but some are attempting, based on printed or Web-based questionnaires, they evaluate the quality of oral or written responses given, of their Internet website or online forums/social media.

To measure the inquirer’s satisfaction is certainly an essential need. The feasibility is debated, as no satisfying method arose: a simple question could be asked to the inquirer at the end of a call; however, this way of assessing the call would certainly lack neutrality. As in most cases no contact details are collected, it is not possible to envision a third person contacting the inquirer back by telephone to measure his/her perception of the conversation. This could be done more easily for the emails. One key strategic question is the added value of the helplines for the patients/inquirers throughout the course of the disease. Also, the inquirers’ category needs further thinking: some groups are largely under-represented (ie, media, psychologists).

For the inquirers’ or patients’ gender we used only three categories: male, female, or unknown. However sexual identity issues exist in rare diseases, with people harboring XXY, XYY, and androgen-insensitivity syndrome. Altogether, sexual abnormalities represented 3.27% (50/1530) of the inquiries concerning rare diseases. We did not integer this characteristic in our survey.

The grouping of rare diseases in 11 categories was an arbitrary process: most of the rare diseases do not belong to one class only as they often are multisystem diseases. In our subgroup of 537 diseases discussed during the inquiries, each rare disease could be classified in three categories on average. For example, Ataxia-telangiectasia (ORPHA code 100, ICD-10 G11.3) belongs to 11 categories in the Orphanet classification.

### Conclusions

Our data suggest helplines, although heterogeneous, are complementary to each other, not competitive. The co-existence of general helplines dealing with all rare diseases and more specific ones benefits the inquirers who can choose which helpline to contact according to the question they have. Inquirers looking for a specialist are often undiagnosed, and will naturally turn to general helplines rather than contacting a specific one, as they do not have a diagnosis yet.

The telephone is still the method of choice to contact a helpline. The impact of the cost for the phone calls was difficult to determine, as only two helplines offered free phone calls to inquirers. The non-free calls were charged as a local call in the vast majority of cases, representing a small expense.

A minimum of 75% of inquiries could be satisfied, within an average of 22.8 minutes, for a number of different rare diseases (536 distinct diseases, including 95 very rare ones). Given the complexity of rare diseases and the scarcity of the information, we consider this outcome as an indicator of a high quality service, to the benefits of the public, and the patients in particular.

Therefore, the service responds to a real demand by the public, however it is not saturated. This leaves the possibility to expand the scope of the helplines, for example, by providing assistance to patients when they are reporting suspected adverse drug reactions as provided for by Directive 2010/84/EU or by providing information on patients’ right to cross-border care, as provided for by Directive 20110/24/EU. The European Network of Rare Diseases Helplines proposes advice and information to guide the creation of helplines where they do not exist yet, as in to estimate the work load, staff, and budget needed.

To make the helplines better known to the public and to increase the European added value of the service, the network asked the European Commission DG Connect to reserve a 116 number for services of social interest. A 116 number is a six digit number, free of charge that can be used by all citizens of the European Union and beyond. In parallel, Member States are developing national plans or strategies for rare diseases and one coordinated objective is to improve information to the public on these diseases. This study demonstrates the helplines’ utility and provides useful information for the planning and budgeting of equivalent services where they do not exist or need to be professionalized.

## Abbreviations

AFM Téléthon: Association Française contre les Myopathies-Téléthon
CVRR: Coordinating Centre Veneto Region
ENERCA: European Network for Rare Congenital Anaemia
ICRDOD: Information Centre for Rare Diseases and Orphan Drugs
MGR: Myasthenia Gravis Romania
MRIS: Maladies Rares Info Services
NORO: Norwegian-Romanian Information Centre
SIO-FEDER: Information and Orientation Service of the Spanish Federation of Rare Diseases
TVMR: Telefono Verde Malattie Rare
VISO: National Organization for Knowledge and Specialist Consultancy

## Multimedia Appendix 1

Data collected from help lines during the survey.
