# Supplementary material for: A European Network of Email and Telephone Help Lines Providing Information and Support on Rare Diseases: Results From a 1-Month Activity Survey
Source: Interact J Med Res. 2014 May 5;3(2):e9. doi: 10.2196/ijmr.2867 (PMC4026574; doi:10.2196/ijmr.2867)
Supplement: Supplementary file 1 [file ijmr_v3i2e9_app1.pdf]

Appendix 1: Type of information requested for the survey

Respondent

Please mention identification for the respondent. This can be a code, not necessarily the name of the person

PS: you can indicate volunteer1, volunteer2, paid staff1, paid staff2 for example

Date of call/email received

Only calls/emails received in November 2012 format: dd/11/2012

Name of disease

Please refer to www.orpha.net codes of rare diseases

Orphanet code

Please refer to www.orpha.net codes of rare diseases

PS: if you have both the code and the name of the disease, please insert a column and use one for the code, one for the disease

Age of inquirer

Please enter the age of the inquirer, not the age of the patient

PS: of course there won't be many enquirers of less than 5-9 years…

0-1
2-4
5-9
10-14
15-19
20-29
30-50
>50
Unknown

Age of patient

Please enter the age of the patient, not the age of the inquirer

0-1
2-4
5-9
10-14
15-19
20-29
30-50
>50
Unknown

Gender of inquirer

Please mention the gender of the person who called/sent an email

Male
Female
Unknown/other

Category of inquirer

PS: the category for partner no longer exists, not with relative or friend

Patient
Children
Relative
Other relative/friend/partner

Professional
Student
Association
Unknown

Type of contact

Please mention if the enquiry was done via one of the following media:
Letter
E-mail
Telephone
Blank
Other

Purpose of contact

Please mention the reason why the person contacted you (several answers possible)
PS1: if several purposes, please create several columns. Please avoid several purposes in same box.
PS2: 2 new categories: info on events, and exemption (to obtain full reimbursement of medical costs)
Information on disease
Specialist/centre
Contact with other patient
Support
Social care
Exemption
Association
Follow-up
Sign-posting
Info on events
Other
Blank

Response given

Several answers possible
PS: if several responses given, please insert columns, do not mention several responses in same box
Provide contact with relevant organisation
Provide information on how to create an organisation
Provide info on disease and care
Provide information on scientific litterature and research
Legal advice
Orientation to expert
Provide information on access to treatment and regulatory affairs
Provide information on cross border care
Psychological support
Provide info on disability/social rights
Contact with other patient
Provide information on clinical trials and registries
Provide information on respite care
Provide info on events
Follow-up
Link to Orphanet or other sites
Blank or other actions

How did the inquirer hear about your help line?

PS: one category suppressed (web)

Relative
Professional
Other persons
Media
Patient org
Flyers & posters
Directories
Other
blank

Region/Country

Please mention the region of inquirer, not the city of residence. If from another country, please mention country and region

Score rating

How do you evaluate the quality of the response you gave? 0 - 10, 10 being the best score

Time taken to manage query from start to finish

How long did the call last for? Or for an email query, how long did it take to reply? (in minutes)

Try to measure the actual duration.
